# Development and In Vitro-Ex Vivo Evaluation of Novel Polymeric Nasal Donepezil Films for Potential Use in Alzheimer’s Disease Using Experimental Design

**DOI:** 10.3390/pharmaceutics14081742

**Published:** 2022-08-21

**Authors:** Paraskevi Papakyriakopoulou, Dimitrios M. Rekkas, Gaia Colombo, Georgia Valsami

**Affiliations:** 1 Department of Pharmacy, School of Health Sciences, National and Kapodistrian University of Athens, 15784 Athens, Greece; 2 Department of Life Sciences and Biotechnology, University of Ferrara, 44121 Ferrara, Italy

**Keywords:** nasal films, nasal delivery, donepezil hydrochloride, permeation profile, design of experiments, Methyl-β-Cyclodextrin, hydroxypropyl-methyl-cellulose, polyethylene glycol, Alzheimer’s disease

## Abstract

The objective and novelty of the present study is the development and optimization of innovative nasal film of Donepezil hydrochloride (DH) for potential use in Alzheimer’s disease. Hydroxypropyl-methyl-cellulose E50 (factor A) nasal films, with Polyethylene glycol 400 as plasticizer (factor B), and Methyl-β-Cyclodextrin, as permeation enhancer (factor C), were prepared and characterized in vitro and ex vivo. An experimental design was used to determine the effects of the selected factors on permeation profile of DH through rabbit nasal mucosa (response 1), and on film flexibility/foldability (response 2). A face centered central composite design with three levels was applied and 17 experiments were performed in triplicate. The prepared films exhibited good uniformity of DH content (90.0 ± 1.6%–99.8 ± 4.9%) and thickness (19.6 ± 1.9–170.8 ± 11.5 μm), storage stability characteristics, and % residual humidity (<3%), as well as favourable swelling and mucoadhesive properties. Response surface methodology determined the optimum composition for flexible nasal film with maximized DH permeation. All selected factors interacted with each other and the effect of these interactions on responses is strongly related to the factor’s concentration ratios. Based on these encouraging results, in vivo serum and brain pharmacokinetic study of the optimized nasal film, in comparison to DH oral administration, is ongoing in an animal model.

## 1. Introduction

The development of advanced drug delivery systems for neurodegenerative diseases has gained great attention, aiming at more effective treatments which are able to overcome the limitations of brain targeting. The main challenge for orally administered drugs is to cross the tightly packed structure of the blood-brain-barrier (BBB). Small and lipophilic drugs can cross the BBB, but their possible binding with serum proteins and/or the first pass metabolism effect significantly decrease the amount of active substance reaching the brain [1]. Consequently, oral route of administration for the treatment of CNS disorders, request to reconcile the physicochemical properties of drugs, with the tight regulation of their movement through the BBB.

The nasal route is a feasible alternative to oral and/or parenteral administration. It is a non-invasive route to achieve nose-to-brain delivery (NBD), local, or systemic action. The nasal administration of radiolabeled proteins in rats indicated the involvement of olfactory and trigeminal nerves in NBD [2,3]. The tight junctions between the olfactory epithelium cells make the permeation rather difficult, but the continuous movement of basal and neuronal cells replacing the nasal mucosa, creating the conditions for greater mucosal permeability. Computational fluid dynamics studies have proven that conventional liquid nasal dosage forms have failed to achieve the drug deposition on the olfactory epithelium [4]. Also, the possible leakage from the nostril or the ingestion of the administered liquid, due to nasopharyngeal communication, cause dose variability and gastrointestinal side effects, respectively. Furthermore, stability issues of the liquid forms and their rapid clearance from nasal mucosa turned scientists’ attention in solid dosage forms (and especially in powders’ formulation). Nasal powders permit the administration of larger doses, able to stick and remain prolonged time on the nasal mucosa. An effort to incorporate advantageous aspects of powders, such as the stickiness and the improved control of deposition site, in liquid dosage forms, is witnessed by the study of nasal gels and in-situ gels [5,6]. 

A versatile dosage form used by many routes of administration such as buccal, ophthalmic, vaginal, and transdermal is the polymer film [7,8,9,10,11]. Contact lenses have been proven effective devices to sustain the release of ophthalmic drugs overcoming the limitations of eye drops, such as the rapid removal from the precorneal cavity due to the tear flow and the nasolacrimal drainage [12]. Since similar limitations should be surpassed in the nasal delivery, the development of circular polymer films with dimensions tailored on those of the olfactory region could increase the residence time in the nasal cavity and the absorption of the film-formulated drugs. Recently, an osmotic nasal polymeric film was developed for topical action in the nasal cavity in early-stage COVID-19 positive symptomatic patients [13]. However, no reports are found on nasal films for systemic and/or NBD delivery. 

The biocompatibility and flexibility/foldability of the films are two critical features for the intranasal application and the tolerability of the formulation in the nostril. Hydroxy-propyl methyl cellulose (HPMC) is a widely used hydrophilic carrier in oral and ocular film development, characterized by high swellability [14]. Moreover, is a widely used excipient in nasal delivery as component of either nasal sprays or mucoadhesive microspheres [15,16,17]. The stability of HPMC in a wide pH range (3–11) and the low possibility of excipient-drug interactions, due to its non-ionic nature, render it the most established excipient in the formulation of hydrophilic dosage forms [18,19]. Furthermore, poly ethylene glycol (PEG), and especially PEG 400, is generally used as plasticizer increasing the structural plasticity and flexibility of polymer films, leading to faster drug release, greater folding endurance, and increased diffusion into the mucus network as well [20,21].

To compensate for the tightness of the nasal mucosa network, as well as the short residence time caused by the mucociliary clearance, permeation enhancers are usually added in the formulation [22]. Until now, two nasal products containing cyclodextrin as excipient have been approved: 1. BaqsimiTM (Eli Lilly, Indianapolis, IN, USA), a glucagone nasal dry powder for the emergency treatment of severe hypoglycemia and 2. Aerodiol^®^ (Servier, Suresnes, France), a nasal spray solution for hormone replacement therapy [23]. According to EMA report on cyclodextrins (CDs) used as excipients [24], CDs have been safely used for nasal delivery at low concentrations (<10% *w*/*v*). The lipophilic β-CD derivative, methyl-beta-cyclodextrin (Me-β-CD), can interact with mucus and loosen the junctions of the barrier enhancing nasal absorption [19]. Moreover, many studies propose the use of Me-β-CD in formulations for neurodegenerative diseases, due to its contribution in depletion of cholesterol [25,26,27].

The development of pharmaceutical dosage forms requires the rational selection of the excipients, in appropriate concentrations to produce a pharmaceutical product able to meet the patient’s needs within the quality by design regulatory framework [28]. Design of experiments (DoE) is the main tool of a statistical thinking approach, and its application aims to elucidate the interactions between the factors which govern a system or a process, leading to the identification of critical process parameters and critical quality attributes, ensuring the quality of the product [29]. DoE is an integral part of pharmaceutical development, strongly recommended by the regulatory authorities (EMA, FDA) [28].

DH is a reversible acetylcholinesterase inhibitor (AChEI) responsible for the restore of acetylcholine levels in the brain. DH oral administration has serious constraints, attributed to its extensive first pass metabolism and flow-dependent entry in the CNS [30], while the need of high doses and the frequent dosing during the day to achieve and retain steady state the drug levels in cerebrospinal fluid, make side effects more intense, thus decreasing the patients’ compliance. Several studies attempt to formulate donepezil in nasal hydrogels to achieve a greater disposition of the drug in the brain [31,32,33]. Nevertheless, the dose measuring remains difficult, as with liquids, causing dose variability and highlighting the need of accurate dose administration [34].

The objective and novelty of the present study is the development and optimization, of an innovative polymer film for potential NBD of Donepezil Hydrochloride (DH), applying DoE. Aiming to introduce a new pharmaceutical dosage form for nasal delivery in AD, that would incorporate critical aspects of the nasal powders, such as the stickiness on the mucosa and the stability, cellulose nasal films with PEG 400 as plasticizer were prepared, containing DH as API (Active Pharmaceutical Ingredient), and Me-β-CD, as permeation enhancer. Nasal films were characterized in vitro and ex vivo and experimental design was used to determine the effects and possible interactions of the selected factors on the permeation profile of DH through rabbit nasal mucosa barrier.

## 2. Materials and Methods

### 2.1. Chemicals and Reagents

Donepezil Hydrochloride (DH, MW: 379.50 g/mol) was supplied by Cipla Ltd. (Mumbai, India) and Methyl-β-Cyclodextrin (Me-β-CD, MW: 1310 g/mol) was purchased by Fluka Chemika (Mexico City, Mexico US & Canada). Hydroxy propyl methyl cellulose (HPMC, Methocel E50 premium LV, MW: 90,000 g/mol) and Poly (Ethylene Glycol) 400 (PEG 400) were purchased from Colorcon (Shanghai, China) and Sigma Chemical Company (St. Louis, MO, USA), respectively. Regenerated cellulose (RC) membranes with MW cut off: 1000 Da and diameter: 63 nm, Dianorm GmbH (Goslar, Germany). Potassium dihydrogen phosphate and Sodium hydroxide 1 mol/L were acquired by Merck KGaA (Darmstadt, Germany) and Chem-Lab NV (Zedelgem, Belgium), respectively. HPLC grade solvents (water, methanol, acetonitrile) and reagents were obtained from Fischer Scientific (Pittsburgh, PA, USA). Triple-deionized water from Fischer Scientific was used for all preparations.

### 2.2. Development of DH Nasal Films

**Figure 1:**
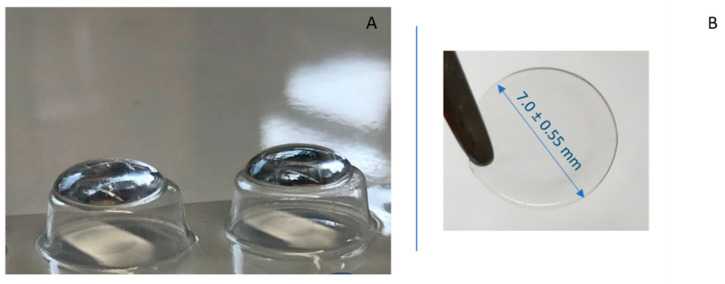
(**A**) 50 μL of the gel placed on the top of cylindrical blisters; (**B**) round, transparent film.

For the development of DH nasal films, HPMC E50 was used as the film-forming polymer in concentrations varying from 1% to 3% *w*/*w*. The process was based on the standard protocol of dispersion of HPMC in hot water (>80 °C), and then hydration at lower temperature (<10 °C) for 15 min [35]. PEG 400 (0% to 3% *w*/*w*) and Me-β-CD (0% to 6% *w*/*w*) were added as film plasticizer and permeation enhancer, respectively, to evaluate their contribution to the film formation and DH transport across rabbit nasal mucosa. More precisely, weighed quantities of HPMC E50 were dispersed in hot distilled water along with PEG 400 and Me-β-CD, under continuous magnetic stirring (600 rpm). Then, the homogenous mixture was cooled to 15 °C to allow for polymer hydration. Weighed amounts of DH were added to the resulting gel, to reach the concentration of 1% (*w*/*w*). Subsequently, 50 μL of the gel were dropped on the top of cylindrical blisters by a Microman E, M250E, 50–250 µL pipette (Gilson, Dunstable, UK), and then let dry for 24 h in room temperature (25 °C). The obtained films (Figure 1) were round and transparent, with diameter equal to 7.0 ± 0.55 mm, containing 0.5 mg of DH as theoretical amount per film. This amount corresponds to 10%, 5% and 2.2% of the recommended oral doses for adults with Alzheimer’s disease (5 and 10 mg/day in mild to moderate cases, and 23 mg/day in severe disease).

### 2.3. Quantitative Analysis of DH

DH was quantified in the prepared films and was determined in the samples of in vitro and ex vivo experiments using HPLC-PDA, a Shimadzu prominence system. The system is composed by a LC-20AD Quaternary Gradient Pump with degasser, with an SIL-HT auto-sampler and a photo-diode array detector SPD-M20A. Data acquisition and analysis were performed by LC solution^®^ software (LabSolutions, version 1.25 SP4, Kyoto, Japan). Analysis was carried out on an analytical reverse phase MZ Analysentechnik Nucleosil 100-5 C18 column (125 × 4.6 mm, 5 μm particle size) connected to a precolumn C-18 (12.5 × 4.6 mm, 5 μm particle size, MZ Analysentechnik) of the same type. Mobile phase consisted of phosphate buffer: Methanol: Acetonitrile (50:30:20) adjusted to pH 2.7 with orthophosphoric acid (80%), in isocratic mode with flow rate 0.8 mL/min. The analysis was performed at 28 °C, the injection volume was 40 μL. The DAD spectra were acquired with 4 nm resolution in the range 200–400 nm and the chosen wavelength was at 268 nm. Bin’ s et al. method [36] was optimized for the needs of the present work and the calibration curve samples ranged from 0.5 μg/mL to 7 μg/mL of DH.

### 2.4. Characterization of DH Nasal Films

#### 2.4.1. Film DH Content

Film DH content uniformity test was performed six times for each formulation (F1-F17 and Film 2), following the method described in the Supplementary Material. For DH assay the method described in the Section 2.3 was applied. The results are expressed as % of the theoretical DH film content (mean ± SD) (see Supplementary Material).

#### 2.4.2. Film Thickness

The thickness of each DH nasal film formulation was measured with INSIZE Outside Micrometer (Suzhou New District, China) with a measuring range of 0–25 mm (0.001 mm graduation). Ten samples of each formulation were tested, and their thickness is expressed as mean ± SD.

#### 2.4.3. Folding Endurance

Film endurance was evaluated by folding the tested round films (mean diameter = 7.0 ± 0.55 mm) repeatedly until they broke. The test was carried out as described by Shinde et al. [37]. The number of folding times until reaching the breaking point are considered as folding endurance value and the test was carried out four times per film formulation and the data were expressed as mean ± SD.

#### 2.4.4. Percent (%) Moisture Loss

Three films of each formulation were weighed and kept in an oven, at 40 °C for 72 h. Weight measurements were taken every 24 h. The following formula (Equation (1)) was used to calculate the percent moisture loss after three days in the oven, where *W_i_* and *W_f_* are the initial and final film weights, respectively [38]. The percent (%) of moisture loss (mean value ± SD) of each formulation is reported in the Supplementary Material.

(1) $$\%\;Moisture\;content=\frac{W_{i}-W_{f}}{W_{i}}\times 100$$

#### 2.4.5. Swelling Test

Three films of each formulation, with mean area 0.4 ± 0.01 cm^2^ were placed on glass slides and 100 μL of phosphate buffer solution with pH 5.6 were added on their surface, to simulate the volume of nasal fluids. After the fluid volume addition, the films were assessed for their ability to be weighted at specific time intervals (5, 15, 30, 60, 120, 180, 240, and 360 s) to calculate the swelling index.

#### 2.4.6. Stability

The manufactured formulations (round transparent films, mean diameter = 7.0 ± 0.55 mm) were stored at 25 °C, in airtight, for six months. The films were packed in nylon PE vacuum bags using vacuum packing machine LAICA VT3205. The appearance and the DH content of the nasal films were macroscopically examined and assayed by HPLC, respectively. The stability study was performed as described in the Supplementary Material. 

### 2.5. DH Release from Films by In Vitro Diffusion Experiments

In vitro release experiments were performed using regenerated cellulose membranes with a molecular cut-off of 1000 Da and Franz-type diffusion cells (Crown Glass, Somerville, MA, USA). After membranes’ pre-treatment and Franz cells’ assembling, as described in a previous work [39], the receptor compartment was filled with 5 mL of PBS (pH = 7.4) and the membrane was mounted between the receptor and donor compartments. The assembled system was allowed to equilibrate at 34 °C for 15 min [40]. Then, a film of each test formulation was placed in the donor compartment and wet with 100 μL of a phosphate buffer solution at pH 5.6. The loading amount of DH in the donor compartment was 0.49 ± 0.01 mg and the area of films (0.38 ± 0.02 cm^2^) covered all of the available diffusion area (0.40 ± 0.05). DH solution (5 mg/mL) in buffer with pH 5.6 was also prepared and tested by introducing 100 μL of the solution in the donor compartment. All experiments lasted for 2 h. At specific time intervals (15, 30, 45, 60, 75, 90, 105, and 120 min after the film placement in the donor compartment), 0.5 mL were sampled from the receptor compartment and replaced by an equal volume of fresh buffer. The samples were analyzed by HPLC [Section 2.3]. At the end of the experiment, the residual formulation in the donor compartment was quantitatively collected and diluted in order to determine the remaining DH and calculate the mass balance. The cellulose membranes were washed with H_2_O/methanol (50:50) solution, to collect the amount of DH remaining in the membrane and the extract was also quantified by HPLC [Section 2.3].

The diffusion area (A) of the Franz cell is equal to 0.636 cm^2^. The flux (*J*) across the artificial membrane to the receptor compartment was calculated from the slopes obtained from regression analysis of the amount of the drug permeated per unit area over the time, according to Equation (2) [41].

(2) $$J=\frac{\mathrm{dQ}}{\mathrm{dt}\times A}$$

### 2.6. Ex Vivo Experiments

Ex vivo mucoadhesive ability and permeation experiments were performed using rabbit nasal mucosa as model barrier [42,43]. Nasal mucosa was extracted on the day of the experiment from rabbit heads collected from a local slaughterhouse (Athens, Greece). Mucosa extraction was carried out according to Manta et al. [44] and the barrier included both the epithelial barrier and the connective tissue. Furthermore, the values of J across the nasal mucosa barrier to the receptor compartment were obtained applying the Equation (3). The apparent permeability (*P_app_*) was calculated by dividing the flux (*J*) by the initial drug concentration in the donor compartment (*C*_0_), as described by the Equation (3) [41]:

(3) $$P_{app}=\frac{J}{C_{0}}$$

#### 2.6.1. Films Mucoadhesive Ability

The mucoadhesive ability of the films was tested on rabbit nasal mucosa tissue positioned on glass slides in 60° degrees angle. The maintenance of the film on the tissue was observed in different time intervals (15, 30, 45, 60, 75, 90, 105, and 120 min after the film placement on the mucosa), respective to that of the permeation study.

#### 2.6.2. Ex Vivo Permeation Experiments

For the ex vivo permeation experiments after the extraction, the mucosa specimen was mounted between the donor and receptor compartments of the Franz cell. Cell equilibration, formulation loading into the donor, sampling, and recovering of residual DH from the donor, were carried out as described in Section 2.5. The entire experiment was performed at constant temperature of 34 °C [45]. The drug accumulated in the tissue was recovered according to the method described by Papakyriakopoulou et al. [39] and then quantified by HPLC, after centrifugation and appropriate dilutions. DH amounts recovered from the mucosa, receptor, and donor compartments allowed for the calculation of the mass balance.

### 2.7. Central Composite Design of Experiments

Response surface methodology was used to determine the optimal nasal film for DH delivery, after a face centered central composite design with three factors at three levels was employed. This method was performed using Design-Expert^®^ v.11 (Stat-Ease, Inc., Minneapolis, MN, USA). The selection of the levels was based on preliminary experiments and the literature [46,47,48]. The chosen factors with their levels, coded as −1 and +1 (low and high level, respectively) and the two responses of the design are presented in Table 1. The design matrix consists of 17 experiments which are shown in Table 2. Folding endurance and permeation across the nasal mucosa barrier were chosen as responses of the applied design after been considered of higher importance for film optimization [21,49]. Specifically, the permeation across the nasal mucosa barrier is a critical parameter for the absorption of DH after the positioning of the film into the nasal cavity, while the folding endurance is considered important for the introduction of the film into the nostril. A non-flexible film will not be able to be inserted and positioned on the olfactory area maintaining its integrity. Possible rupture of the film during administration would lead to dose variability. Consequently, the maximization of these two responses during the optimization is requested to ensure the mechanical stability of the final film.

### 2.8. Statistical Analysis

The design space was constructed and analyzed using the Design-Expert^®^ Software, v.11 (Stat-Ease, Inc., Minneapolis, MN, USA). Analysis of variance (ANOVA) was applied to test the chosen model and individual terms in the model. Concerning the in vitro release and ex vivo permeation experiments, data distribution was tested using the Shapiro-Wilk (S-W) normality test. Significance was set at *p* < 0.05 level and all tests were two-tailed with 95% Confidence Intervals (CI). Results are expressed as mean ± SD for the in vitro release experiments and mean ± standard error (SE) for ex vivo permeation experiments. The permeation values were statistically compared between the different formulations and per timepoint within the formulation. Outlier detection occurred applying the Interquartile Range (IQR) using a step of 1.5 × IQR. No outliers were detected. Normality test indicates a non-Gaussian distribution of the data. Hence, non-parametric tests were applied for the data of both in vitro and ex vivo experiments. Kruskal-Wallis was performed to statistically evaluate the differences between the formulations at every time point of the experiment and post-hoc Mann-Whitney to detect individual differences. Data analysis was performed using SPSS version 26.0 (IBM SPSS Statistics for Windows, Version 26.0, IBM Corporation, Armonk, NY, USA) software package.

## 3. Results

In the following sections, we present the results from the in vitro and ex vivo characterization of the DH nasal films, after the application of the central composite experimental design. More specifically the prepared formulations were characterized in terms of drug content uniformity, film thickness, folding endurance, % moisture loss, and DH permeation across artificial and biological barrier. Film thickness and the percentage of moisture loss were not measured for formulations F8 and F17, as no film was formed due to the actual composition. 

### 3.1. Characterization of DH Nasal Films

#### 3.1.1. Film DH Content

DH content ranged from 90.0% to 99.8% (0.45 to 0.5 mg of DH, respectively) of the theoretical loading dose (observed difference less than 10%), and standard deviations varied from 4.9% to 1.6%. The calculated acceptance values (%) were below 15%, ranging from 8% to 14% [50]. For further information, please refer to the Supplementary Material.

#### 3.1.2. Film Thickness

Thickness measurements of nasal film range from 19.6 ± 1.9 μm (F5) to 170.8 ± 11.5 μm (F2) with % RSD < 10%. The thickness of the manufactured formulations is correlated with the concentration of the three components in the films. More precisely, as it is expected, increasing the concentration of polymer and/or plasticizer and permeation enhancer, the film thickness increases as well. According to the limits set, all of the films were considered accepted in terms of thickness. Further information, concerning the thickness limits, are included in the Supplementary Material.

#### 3.1.3. Folding Endurance

**Figure 2:**
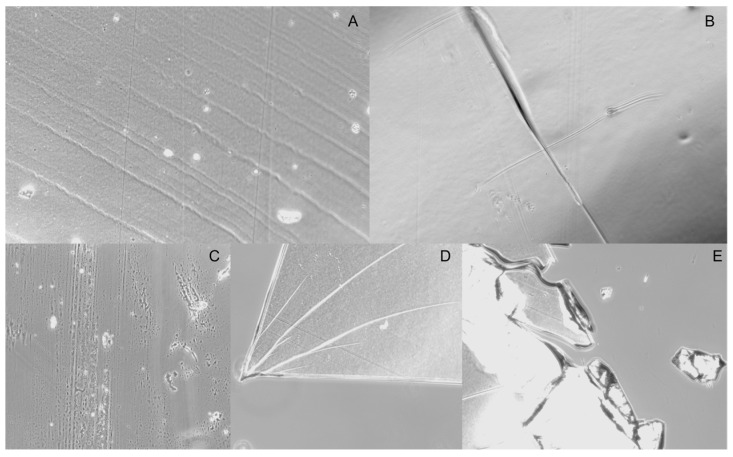
(**A**) F1 film surface before folding, (**B**) folding line without signs of crack on F1 surface, (**C**) F6 film surface before folding, (**D**) crack on F6 surface after one-fold-time, (**E**) F6 film fragments after cracking.

To evaluate the flexibility of the prepared round films, they were repeatedly folded and unfolded along the same line. The Standard Test Method for Flexibility Determination of Films by Mandrel Bend (D 4338-97) was modified and adjusted for small size nasal films. According to the test, the film is defined flexible if no crack is visible, using the Olympus CKX41 microscope with the Moticam Pro imaging camera, in 10× magnification [51]. In this study, the films folded 180° along the same line for five times, without any signs of crack, were considered the most flexible. In Figure 2, images of F1 and F6 films before and after folding, were obtained using the Motic Images Plus 2.0^®^ software (Moticseries, Barcelona, Spain), as representatives of the presence or absence of cracks.

The results in Table 3 show that the presence of Me-β-CD (factor C) at its high level (6% *w*/*w*), decreased the flexibility of the film, with the reduction becoming more evident in the absence of plasticizer (PEG 400) and at the lower level of factor A (HPMC E50) (F_6_ < F_4_ < F_2_ < F_14_). Furthermore, at the high level of factor C, it was not possible to form a film that could be detached from the blister, in particular when the three ingredients coexisted in the formulation and the C:A ratio was greater than 2, as in the case of F_8_ (A:B:C = 1:3:6) and F_17_ (A:B:C = 2:1.5:6). Formulations containing Me-β-CD in concentration ≤3% *w*/*w* were proven highly flexible, as they bore four and five folds. 

#### 3.1.4. Percent (%) Moisture Loss

The tested formulations showed a moisture loss in the range of 0.8–3%, indicating the robustness of manufacturing process and of the resulted films, which corresponds to the residual water in the pharmaceutical product. However, the determination of % moisture loss was not possible in the case of formulations F8 and F17 as no film was formed (as previously mentioned).

#### 3.1.5. Swelling Test

**Figure 3:**
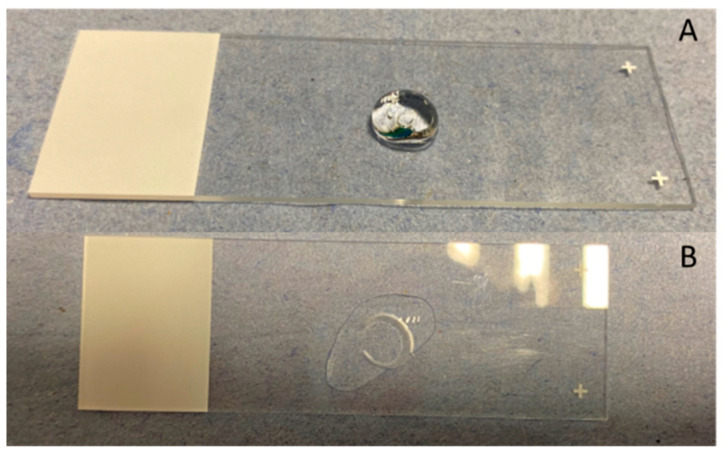
Films’ swelling after the addition of 100 μL of phosphate buffer solution with pH 5.6 at the time point of (**A**) t = 0 s and (**B**) t = 5 s, after the addition of the solution.

The addition of 100 μL of phosphate buffer solution (pH 5.6) on each film resulted in the immediate conversion of every tested formulation in gel. This conversion took place in less than 5 s for all of the tested formulations. Consequently, it was not possible to calculate the swelling index as the weight measurements of the films after the addition of the 100 μL were not able to be performed. In Figure 3, the formulation corresponding to the center point of the design space (F7, F10, F11) is shown as representative of the performance of all films.

#### 3.1.6. Stability

The appearance of the films after six months of storage remained unchanged. After this period, the mean DH content of all formulations, apart from F2 and F12, ranged from 90–105% of the initial content (day 0). In contrast, the DH content of formulation F2 and F12 decreased to 74% ± 13.4 and 77% ± 4.6, respectively. The hypothesis of a particular DH interaction with the three components of the formulation, which are included at their higher values in this formulation, may be a possible explanation for the incomplete and highly variable (20.5 and 18.1% RSD, at three months and six months, respectively) DH quantification in the nasal film after the period of three months. However, further research is needed, to map the occurring molecular interactions between the three components and the API, the existence of which is also indicated by the results of the experimental design.

The individual mean values of DH content (expressed as % of the initial content) of each formulation, at the time points of the stability study (one, two, three, six months) and further discussion are presented in the Supplementary Material.

### 3.2. DH Release from Films by In Vitro Diffusion Experiments

**Figure 4:**
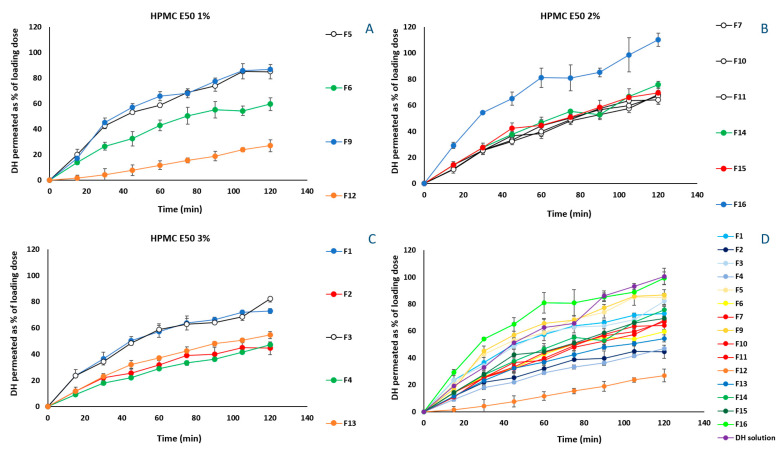
Amount released for formulations containing 1% HPMC E50 (**A**), 2% HPMC E50 (**B**) and, 3% HPMC E50 (**C**), and for all tested formulations in comparison to the DH solution (**D**), expressed as % of loading dose (mean ± SD, n = 3).

In vitro release experiments of DH from the 17 manufactured formulations, were performed using diffusion Franz cells and regenerated cellulose membranes with defined pore sizes as diffusion barrier. The molecular cut-off of 1000 Da enables the permeation of free DH, blocking that of the excipients and/or of the possible derived complexes, in case of interactions occurring among the components of the formulation. The amount diffused is expressed as percentage of the loading dose. DH is a freely soluble compound and therefore, no differences are expected among the release profiles of the prepared formulations. However, in Figure 4A–C, it is observed that the formulations without Me-β-CD (Figure 4A: F5, F9; Figure 4B: F16, Figure 4C: F1, F3) performed better and DH release values ranged from 72.9 ± 2.0% to 110.3 ± 5.1%.

The application of Equation (2) allows the calculation of the flux for each prepared formulation, which varies from 4.4 ± 0.063 to 2.3 ± 0.024 μg/cm^2^/min. Moreover, the R-square values reveal the first-order release for all of the tested formulations. The flux values of each formulation are included in Table S4 of the Supplementary Material. Moreover, the quantification of DH remaining in the cellulose membrane revealed that 1.6 ± 0.71% of the loading doses is retained on average by the artificial membrane. Membrane retention data of each formulation expressed as the percent (%) of the loading dose retained by the cellulose membrane ± SD, are included in Table S6 of the Supplementary Material.

### 3.3. Ex Vivo Experiments

#### 3.3.1. Films Mucoadhesive Ability

**Figure 5:**
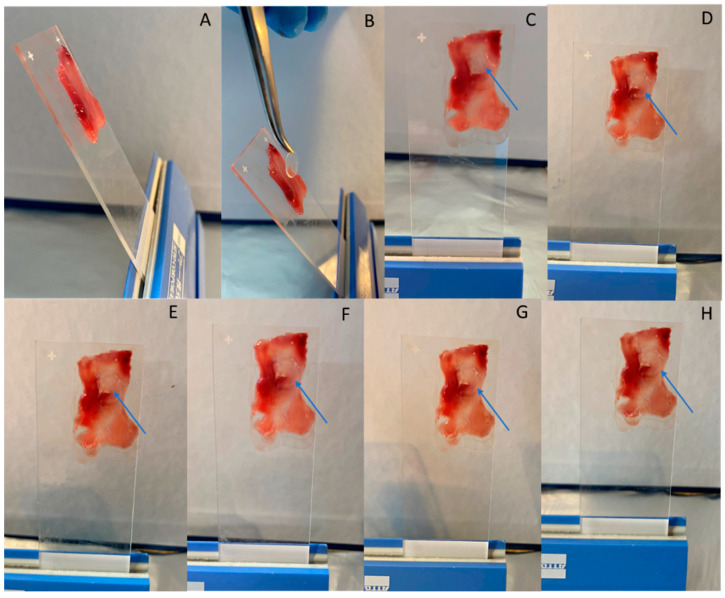
Evaluation of films’ mucoadhesive ability on nasal mucosa tissue positioned on glass slides in 60° degrees angle: (**A**) nasal mucosa, (**B**,**C**) Film positioning on nasal mucosa (blue arrow) at the time point of t = 0 min, (**D**) t = 30 s, (**E**) t = 5 min, (**F**) t = 30 min, (**G**) t = 60 min, (**H**) t = 120 min, after the positioning.

The assessment of the mucoadhesive ability of the films on the nasal mucosa showed that all of the prepared films (F_1_–F_17_) remained stationary on the placement site for 2 h. The structure of each film was incorporated into the tissue, forming a stable jelly circle on the mucosa surface in less than 5 s after the positioning. In Figure 5, the formulation corresponding to the center point of the design space (F_7_, F_10_, F_11_) is shown as representative of the performance of all films. 

#### 3.3.2. Ex Vivo Permeation Experiments

**Figure 6:**
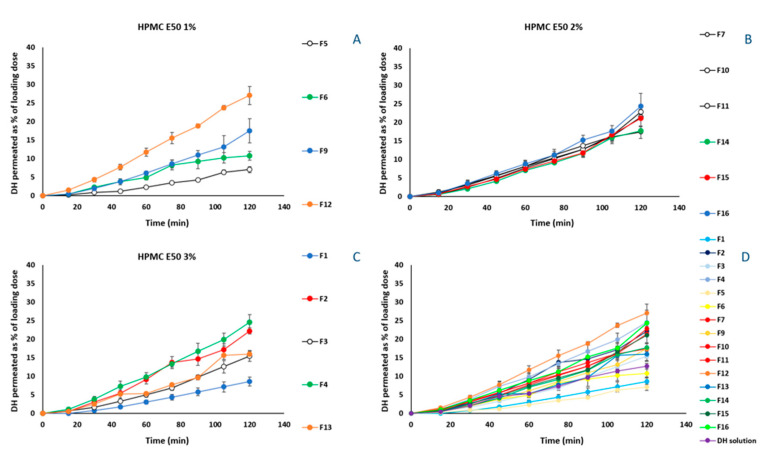
Permeation profiles through rabbit nasal mucosa for formulations containing 1% HPMC E50 (**A**), 2% HPMC E50 (**B**) and, 3% HPMC E50 (**C**), and for all tested formulations in comparison to the DH solution (**D**), expressed as % of loading dose (mean ± SΕ, n = 3).

The results of ex vivo permeation experiments are presented in Figure 6. Differently from the profiles obtained from the in vitro experiments, in Figure 6A, the permeation profile of F5 (1% HPMC E50), which permeated significantly less (*p* < 0.05) across the rabbit nasal mucosa, proves the contribution of Me-β-CD and PEG 400 to DH permeation, when HPMC E50 is present at its lowest level. Specifically, the presence of either Me-β-CD (F6) or PEG 400 (F9) increased similarly the amount of DH crossing the barrier until the 90 min. Thereafter, the effect of PEG 400 resulted in greater permeation at 120 min (17.5 ± 3.2%), than that derived from Me-β-CD effect (10.8 ± 1.2%). The positive effect of the presence of hydrophilic molecules, such as PEG 400, in HPMC formulations has been extensively studied. Specifically, PEG 400 used as plasticizer, increases the segmental mobility of HPMC E50 polymer chains, increasing the number and size of the available diffusion channels as well [52]. In addition, Me-β-CD acting as permeation enhancer interacts with the mucosal epithelium influencing the integrity of the barrier, eventually increasing DH permeability. The formulations containing 1% HPMC E50 and both the permeation enhancer and the plasticizer (F9, F12), presented the greatest permeation (Figure 6A), implying the positive effect of the three-component interactions. 

Nevertheless, when HPMC E50 was present at a 2% concentration (F7, F10, F11, F14–F16), no significant differences (*p* > 0.05) were observed among the formulations, at all of the time points. In Figure 6C, where showing permeation profiles for the formulations containing 3% of HPMC E50, it is noted that the contribution of the permeation enhancer is more pronounced. 

Among all of the tested formulations F12 exhibited the most preferable permeation characteristics (Figure 6D). However, the obtained film presents a quite sticky nature, which renders it difficult to handle, and possibly to be placed in the nasal cavity. 

The flux across the nasal mucosa barrier for all of the tested formulations varies from 0.26 ± 0.038 to 1.86 ± 0.040 (μg/cm^2^/min), while the apparent permeability ranges from 4.18 × 10^−4^ to 0.53 × 10^−4^ (cm/min). The values of flux and apparent permeability for each formulation are included in Table S5 of the Supplementary Material.

The quantification of DH remaining in the nasal mucosa barrier revealed that 10.1 ± 4.90% of the loading doses is retained on average by the biological membrane. Membrane retention data of each formulation expressed as the percent (%) of the loading dose retained by the nasal mucosa barrier ± SD, are included in Table S6 of the Supplementary Material.

### 3.4. Central Composite Design of Experiments

ANOVA table (Table 4) for the DH amount permeated at 60 min (Y_1_), expressed as % of the loading dose, revealed a reduced cubic model as significant (R^2^ = 0.8885, F = 25.24, *p* < 0.0001), with non-significant lack of fit (*p* > 0.0001). Moreover, the predicted R^2^ agreed with the adjusted R^2^ presenting a difference less than 0.2. Regarding the independent variables of the reduced cubic model, factors A, C and the interactions, AC and ABC were found significant (*p* < 0.0001). Also, the second-order effect of factor Me-β-CD (C^2^) and the interactions A^2^C and AB^2^ were also significant parameters as witnessed by the high F-values (varying from 48.59 to 78.49) and the low *p*-values (*p* < 0.0001). 

**Figure 7:**
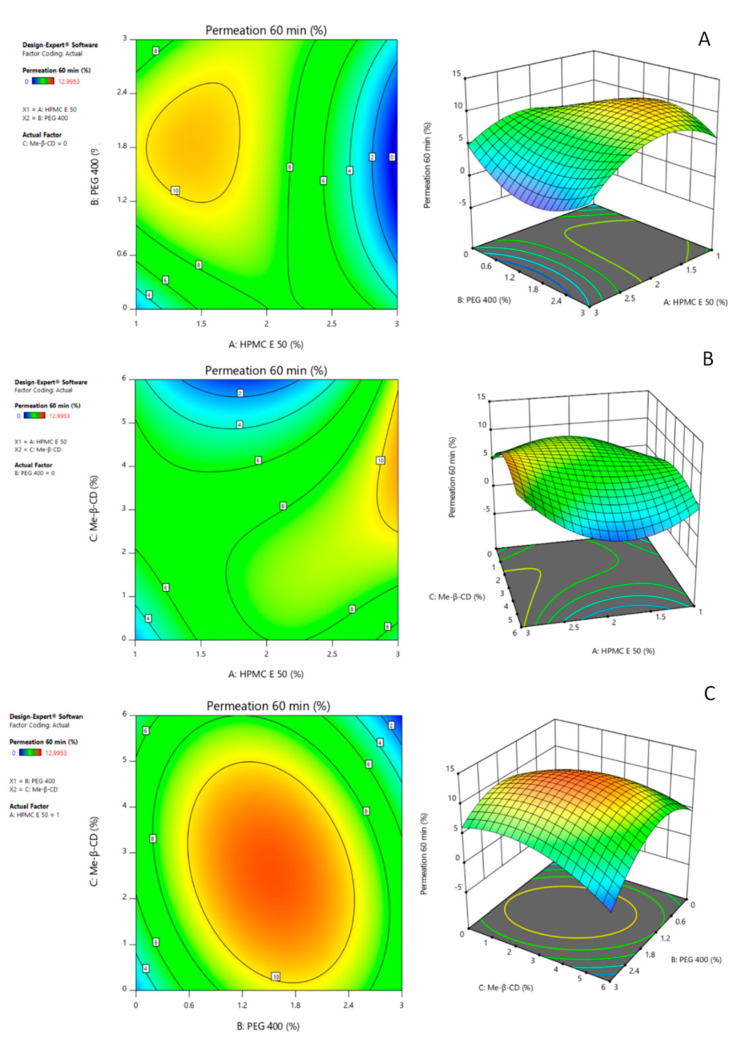
Effect of interactions (**A**) HPMC E50–PEG 400, (**B**) HPMC E50–Me-β-CD, (**C**) PEG 400–Me-β-CD, when HPMC E50 was kept constant at 1% *w*/*w*, on the permeation of DH across the rabbit nasal mucosa barrier, represented by the 2D contour plots (**left** panel) and 3D surface plots (**right** panel).

To understand the three-factor interaction ABC, as well as the mixed terms A^2^C and AB^2^, 2D contour and 3D surface plots were employed. In Figure 7A, showing the effect of the interaction between factors A, B on response Y_1_, it is noted that Y_1_ is maximized when HPMC E50 and PEG 400 are presented in concentrations equal to 1.5% *w*/*w* and 1.8% *w*/*w*, respectively. In the contrast, when factor A reaches its maximum value, regardless of the value of factor B, response Y_1_ decreases. Concerning the interaction AC, as evident also from Figure 7C, the positive effect of the permeation enhancer is more expressed when factor A is at its highest value. More precisely, Y_1_ is maximized when the concentration of HPMC E50 is 3% (*w*/*w*) and of Me-β-CD varies from 3 to 5% (*w*/*w*). The lowest DH permeation across the rabbit nasal mucosa is noted when factor C reaches its maximum value and factor A varies from 1.5 to 2% *w*/*w* (Figure 7B). The three factors interaction ABC leads response Y_1_ to maximization around the intermediate values of factors B and C, when formulation contains 1% (*w*/*w*) of HPMC E50. The model fits to the data managing to describe the positive effect of the three-component interaction.

Regarding the response Y_2_, corresponding to the folding times of the prepared films, ANOVA table (Table 5) suggested the reduced quadratic model as significant (R^2^ = 0.9513, F = 88.98, *p* < 0.0001), with non-significant lack of fit (*p* > 0.0001). The factor C, as well as its second-order effect C^2^ and its interaction with factor A (AC), were found to be significant (*p* < 0.0001). Factor B, its interactions with the other factors, and the second-order terms A^2^, B^2^, were excluded as non-significant terms of the system. In addition, the predicted R^2^ agrees with the adjusted R^2^ presenting a difference less than 0.2. 

**Figure 8:**
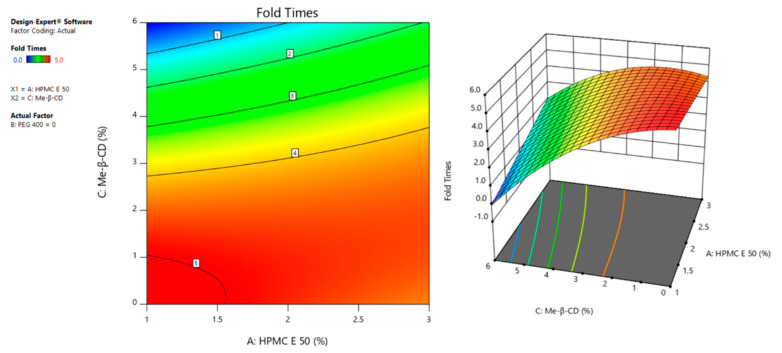
Effect of the interaction HPMC E50–Me-β-CD, on the folding endurance of the films, represented by the 2D contour plot (**left** panel) and 3D surface plot (**right** panel).

The high flexibility/foldability, expressed by 4 and 5 consecutive folds without cracks, of the films containing Me-β-CD in concentration ≤3% *w*/*w*, can be attributed to the positive effect of AC interaction, which is compensated by the negative effect of factor C, when it is at its high level. In 2D contour and 3D surface plots (Figure 8), it is evident that, regardless of the concentration of HPMC E50, the presence of factor C, at values up to 2.5% *w*/*w*, leads to the formation of flexible films.

### 3.5. Optimization of DH Nasal Films

For the optimization of DH nasal films, two candidate optimum formulations were prepared and evaluated. More precisely, desirability function was applied either by setting all of the factors to be in range (Film 1), or by setting the value 1.5 as the lower limit of factor A (Film 2). In both cases the maximization of the two responses Y_1_, Y_2_ was selected, and the importance of each response was rated with 5. The composition of the suggested optimum formulations, the desirability, as well as the predicted values for the two responses are presented in Table 6. The increase of the lower limit of factor A aimed to the preparation of a film with easier handling, after experiencing the difficulty to detach Film 1 from the blister. In vitro and ex vivo evaluation of Films 1, 2 resulted in 38% and 10% deviation between the predicted and observed values, respectively, for response Y_1_, while for response Y_2_, the deviation was 0%, for both films. The higher mean value of % of loading dose permeated at 60 min and the significantly lower deviation between the predicted and observed value for response Y_1_, in the case of Film 2, led to the selection of this composition as the optimum nasal film. 

**Figure 9:**
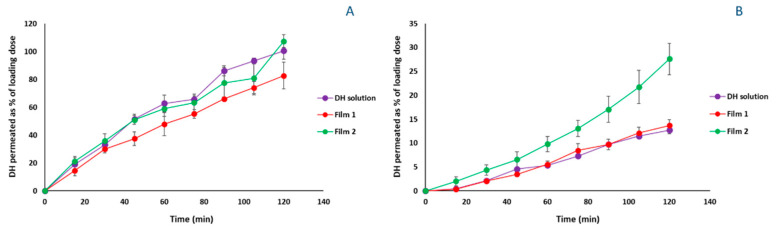
(**A**) Amount released and (**B**) permeation profiles for Films 1,2, in comparison with the DH solution, expressed as the % of loading dose (mean ± SD and mean ± SE, respectively, n = 3).

In vitro release experiments showed that the release profile of Film 2 was linear (R^2^ = 0.9464, Table 7) and equal to that of the DH solution (Figure 9A), while ex vivo experiments showed that the permeation of % loading dose at 60 min was equal to 9.79%, reaching the value of 27.62% at the time point of 120 min (Figure 9B). The permeation of the DH across the nasal mucosa barrier at the time point of 120 min, in the case of Film 2, found to be twice higher than that resulted from the Film 1 and the DH solution. Moreover, the value of flux (*J*) and apparent permeability (*P_app_*) of Film 2 are 1.5 times higher than that of Film 1 (Table 7).

## 4. Discussion

The BBB is a peculiar organization of the CNS microvasculature, compromising endothelial cells with no fenestrations and extensive tight junctions. The capillary basal lamina, astrocytes and pericytes embedded within the basal lamina complete its structure [53]. It is a strong biological barrier which impedes molecules’ free penetration into the brain. Olfactory and trigeminal pathways link the nasal cavity with the CNS, bypassing BBB and rendering brain parenchyma accessible to wide range of APIs. The olfactory pathway allows to target the rostral area of the brain [54]. Conversely, through the trigeminal pathway, the drugs can be transferred to brainstem either to caudal or rostral parts [5]. The objective of nasal products evolution is to achieve therapeutics drug levels in brain, taking advantage of the NBD. Specifically, the development of nasal films for NBD of CNS drugs aims to surpass the limitations of oral administration, or those related to the formulation of these medication in nasal sprays or powders. The first attempt to formulate nasal adhesive patches intended to produce a topical formulation for the treatment of dry nasal syndrome [55], while recently an osmotic nasal surface cleaning, virus, and cytokine trapping polymeric film was developed for topical action in the nasal cavity in early-stage COVID-19 positive symptomatic patients [13]. However, no reports are found on nasal films for systemic and/or nose-to-brain drug delivery. A sophisticated approach towards the formulation factors should be established considering the effective positioning near the olfactory region in the nasal cavity and the improve the contact with the nasal mucosa tissue.

In the present study, round, transparent films were designed and evaluated in terms of their quality attributes to determine the optimum combination of factors A, B, and C for the development of an innovative dosage form, able to be administered intranasally. The prepared nasal films are characterized by good uniformity of DH content (>90.0%) and appropriate thickness to be placed in the nasal cavity without cause any breathing obstruction [56,57]. 

Concerning the components of the films, HPMC is a hydrophilic polymer, widely used for the preparation of film formulations [58,59]. HPMC molecules tend to intertwine with the mucin chains forming hydrogen bonds, thereby giving to the film formulation some mucoadhesion properties [60]. This was also confirmed by the determined film’s mucoadhesive ability (Figure 5). This interaction is favored by the presence of hydroxyl groups and can also be affected by the wetting and the swelling of the polymer. Also, the low viscosity derivatives, such as HPMC E5, E15, and E50, are preferable, being characterized by favorable dissolution profiles attributed to the looser links between the polymer molecules [61].

In this study, HPMC E50 (Factor A) was the selected polymer for film formation in concentration range from 1% to 3% *w*/*w*. The in vitro release profiles of F3 and F5 (82.2 ± 1.5% and 85.0 ± 5.7%, respectively, at the time point of 120 min), containing only HPMC at the highest and lowest level, respectively, prove that the concentration of HPMC did not affect the release of DH (Figure 4D, *p* > 0.05). Nevertheless, its involvement with the mucus layer differentiates the permeation of F3 and F5 through the rabbit nasal mucosa barrier, indicating the superiority of F3 (Figure 6D). The significance of variation of HPMC E50 concentration in DH permeability through the biological barrier (response Y1) is also described by the reduced cubic model of the central composite design. Moreover, the experimental design revealed the significance of AC (HPMC, Me-β-CD) and ABC (HPMC, PEG400, Me-β-CD) interactions. More precisely, in the case of F2 and F4 formulations, the interaction of Me-β-CD with the nasal epithelium, can increase the paracellular permeability and loosen the junctions of the barrier rendering it more permeable [62]. On the contrary, from formulation F1, which does not contain Me-β-CD, DH permeates less the rabbit nasal mucosa. Furthermore, the greater permeation of F3 (HPMC E50 3%) compared with F1 (HPMC E50 3%, PEG 400 3%), implies a negative effect of PEG 400 on DH permeability, when HPMC E50 takes its highest value. However, this effect does not depend on the HPMC E50-PEG 400 interaction as per Figure 5C, wherein the performance of formulations F1, F3 was the same. Consequently, it is probably due to the involvement of mucus into the network of HPMC E50-PEG 400, when the two polymers are at the highest concentration (3%). Hence, the interaction of the two macromolecules leads to the formation of a dense polymeric-chains-network, in which glycosylated proteins of the mucus layer are probably also involved [63]. The positive effect of PEG 400 on permeation of DH, in the case of 1% HPMC E50 films is probably eliminated at the high levels of HPMC, by the concurrent bonding of PEG molecules with the sugar moieties of either the HPMC or the glycosylated proteins.

The results allow one to hypothesize the formation of an HPMC- Me-β-CD network, which results in films with higher flexibility, indicating the positive effect of AC interaction in response Y2 (Fold times). However, at higher concentration of factor C and lower concentration of factor A, the interaction acts negatively on response Y2 (Figure 8), either decreasing the flexibility (F6) or impeding film formation (F8, F17). 

In vitro release experiments revealed that the DH release profiles of formulations F3, F5, F9, and F16 did not significantly differ (*p* > 0.05) from that of DH solution (5 mg/mL, pH 5.6). The composition of these films has as common feature the absence of Me-β-CD, indicating the possible complexation of DH by CD molecules, fact that delays DH diffusion through the artificial membranes. Specifically, the formulations containing Me-β-CD demonstrate a lower cumulative amount diffused. The formation of intermolecular bonds between DH and Me-β-CD could be hypothesized, probably involving also HPMC molecules, in these interactions. If a DH-Me-β-CD complex was formed, it could impede the diffusion of DH, since only the free drug can cross the artificial membrane barrier. The inclusion of donepezil free base into the cavity of HP-β-CD has been investigated with FT-IR and molecular modeling [47]. However, the addition of the DH salt and the incorporation of a more lipophilic β-DC derivative, in the case of this study, requires further research of the possible inclusion or non-inclusion interactions. Regarding the presence of PEG 400, it did not present any effect (positive or negative) on DH diffusion though the artificial membrane witnessed by the profiles of F5, F9 (Figure 4A) and F1, F3 (Figure 4C). 

Concerning the interaction ABC, to our knowledge, there are not relevant studies mapping the bonds and forces that may be developed among these three excipients. However, in an attempt to approach this interaction, the ability of CDs to form ternary complexes with drugs and HPMC or PEG [64] has been described extensively in literature, especially for their greater solubilizing effect compared with that of binary CD/drug complexes [65]. Also, the entanglement of HPMC molecules in the CD-drug network acts as release modulator rendering possibly the development of sustained released formulations containing both polymers [66]. In addition, PEG entrapment in CD inner cavity has been proven in case of α-CD but is disputed in the case of β-CD due to the difficulty of the polyether to fit in a cavity of greater dimensions [67]. However, PEG’s hydrogen-bonding capability favors its interlinkage with either the inner surface of the Me-β-CD truncated cone, or with the hydrogens of methyl groups of the outer surface. Thus, the Me-β-CD molecules may be cross-linked and been arranged in polymeric configuration. Furthermore, plasticizers and namely, low molecular weight PEG molecules (e.g., PEG 400, 600), can interpose in the polymer network of HPMC, forming hydrogen bonds with the functional groups of the polymeric chain. The effect of ABC interaction is maximized when factors B, C approach their center values and factor A takes its lower value, as shown by the curvature of 3D surface plot in Figure 7C. When the concentration of factor A increases, lower CD concentrations are required to achieve Y2 maximization.

The significance of ABC interaction is also depicted in the results of the optimization process. Aiming to maximize both responses (Y1, Y2), the chosen models propose the involvement of all three factors. The constrain set in concentration of factor A aims to solve the problem of sticky film arising by the composition of Film 1. In addition, regarding response Y1 in confirmation experiments, the % error in the case of Film 2 was 10%, and significantly lower (*p* > 0.05) compared to Film 1.

## 5. Conclusions

In the present work, biocompatible polymeric, HPMC-based nasal films for drug delivery were developed, for the first time employing PEG 400 as plasticizer agent and Me-β-CD as a permeation enhancer. Films were tested in vitro and ex vivo for their formulation characteristics and nasal mucosa permeability, respectively. The application of face centered central composite design with three factors at three levels revealed the composition which ensure the formation of a flexible film and the maximization of DH permeation. It was found that all of the selected factors interacted with each other in multiple ways, and the cumulative effect of these interactions on responses is strongly related to the concentration ratios among the factors. The results presented are quite encouraging and, to this end, in vivo pharmacokinetic studies in appropriate animal model are ongoing to evaluate the performance of Film 2 for systemic and NBD, compared to DH oral administration. In addition, further research is in progress to decode the binding forces developed between the excipients and the API, and how these forces may alter film formation characteristics, as well as DH permeation through the biological barriers.

## Figures and Tables

**Table 1:** Factors, levels, and responses of the experimental design.

	Factors	Units	Low Level (−1)	Intermediate Level (0)	High Level (+1)	Response 1 (Y1)	Response 2 (Y2)
A	HPMC E50	% (*w*/*w*)	1	2	3	% of the loading dose permeated after 60 min	Folding times
B	PEG 400		0	1.5	3		
C	Me-β-CD		0	3	6		

**Table 2:** Composition of the film-forming formulations derived from the central composite experimental design.

Formulation	HPMC E 50		PEG 400		Me-β-CD	
	% *w*/*w*	Coded Value	% *w*/*w*	Coded Value	% *w*/*w*	Coded Value
F_1_	3	1	3	1	0	−1
F_2_	3	1	3	1	6	1
F_3_	3	1	0	−1	0	−1
F_4_	3	1	0	−1	6	1
F_5_	1	−1	0	−1	0	−1
F_6_	1	−1	0	−1	6	1
F_7_	2	0	1.5	0	3	0
F_8_	1	−1	3	1	6	1
F_9_	1	−1	3	1	0	−1
F_10_	2	0	1.5	0	3	0
F_11_	2	0	1.5	0	3	0
F_12_	1	−1	1.5	0	3	0
F_13_	3	1	1.5	0	3	0
F_14_	2	0	0	−1	3	0
F_15_	2	0	3	1	3	0
F_16_	2	0	1.5	0	0	−1
F_17_	2	0	1.5	0	6	1

**Table 3:** Fold times of the prepared films.

Fold Times	Formulation
0	F_6_, F_8_, F_17_
2	F_4_
3	F_2_
3.5	F_14_
4	F_3_, F_7_, F_10_, F_11_, F_13_, F_15_
5	F_1_, F_5_, F_9_, F_12_, F_16_

**Table 4:** Independent factors for the estimated effects, F-values, and associated *p*-values for response 1 (% of loading dose permeation after 60 min), after the analysis of variance.

ANOVA for Reduced Cubic Model	F-Value	*p*-Value	
Model	25.24	<0.0001	significant
A-HPMC E 50	35.24	<0.0001	
B-PEG 400	1.85	0.1817	
C-Me-β-CD	67.33	<0.0001	
AB	0.5103	0.4794	
AC	44.42	<0.0001	
BC	12.08	0.0013	
A^2^	3.34	0.0756	
B^2^	0.8244	0.3696	
C^2^	48.59	<0.0001	
ABC	20.84	<0.0001	
A^2^C	78.49	<0.0001	
AB^2^	66.66	<0.0001	

**Table 5:** Independent factors for the estimated effects, F-values, and associated *p*-values for response 2, after the analysis of variance.

ANOVA for Quadratic Model	F-Value	*p*-Value	
Model	88.98	<0.0001	significant
A-HPMC E 50	14.64	0.0004	
B-PEG 400	10.16	0.0027	
C-Me-β-CD	587.08	<0.0001	
AB	8.13	0.0068	
AC	73.18	<0.0001	
BC	0.0000	1.0000	
A^2^	15.61	0.0003	
B^2^	0.9989	0.3235	
C^2^	85.57	<0.0001	

**Table 6:** Composition of the two candidate optimum formulations; model desirability; predicted and observed values of responses Y1,2.

Optimization		A	B	C	Desirability	Permeation of % Loading Dose 60 min (Y_1_)	Fold Times (Y_2_)
		% *w*/*w*					
Film 1	Prediction	1	1.59	1.95	0.954	11.83 ± 0.72	5
	Observation					7.36 ± 1.25	5
Film 2	Prediction	1.5	1.7	0.8	0.927	10.91 ± 0.52	5
	Observation					9.79 ± 1.57	5

**Table 7:** The flux across the cellulose membrane (J_CM_) (mean ± SD, n = 3), the flux (J_NM_) mean ± SD, n = 3) and the apparent permeability (*P_app_*) across the nasal mucosa barrier of Films 1,2. R-square of regression analysis of the amount of the drug permeated per unit area over the time, across the cellulose membrane and the nasal mucosa barrier are included in the table [R^2^ (CM) and R^2^ (NM), respectively].

Formulation	J_CM_ (μg/cm^2^/min)	R^2^ (CM)	J_NM_ (μg/cm^2^/min)	R^2^ (ΝΜ)	*P_app_* (cm/min) × 10^−4^
Film 1	4.60 ± 0.015	0.9934	1.22 ± 0.063	0.9840	2.59
Film 2	5.79 ± 0.056	0.9464	1.82 ± 0.000	0.9740	3.71

## Data Availability

Not applicable.

## Supplementary Materials

The following are available online at https://www.mdpi.com/article/10.3390/pharmaceutics14081742/s1, Figure S1: Thickness (mean ± SD, n = 10) of manufactured nasal films as a function of the % concentration of HPMC E50 in the film-forming solution. Data labels indicate the % of PEG 400 and/or Me-β-CD, in the formulation, Figure S2: Stability profiles (mean ± SD, n = 3) of the 6 month-stability study performed, in airtight packaging, in room temperature, on formulations F1–F17 and optimized formulation Film 2, Table S1: Film weight and content uniformity measurements expressed as % of the theoretical DH film content (mean ± SD, n = 6), Table S2: Nasal film storage stability results, at 25 °C, presented as relative to day 0 % mean DH amount ± SD (n = 3), Table S3: Percent (%) moisture loss of the formulations F1–F17 and optimized Film 2 (mean ± SD, n = 3), Table S4: Flux (J) (mean ± SD, n = 3) and R-square of the regression analysis of the amount of the drug permeated per unit area over the time, for formulations F1–F7, F9–F16, and Film 1–2, Table S5: Flux (J) (mean ± SD, n = 3), R-square of the regression analysis of the amount of the drug permeated per unit area over the time and the apparent permeability (Papp) across the nasal mucosa barrier, for formulations F1–F7, F9–F16, and Film 1,2, Table S6: Percent (%) of the loading dose retained by the cellulose membrane and the nasal mucosa barrier, of the formulations F1–F7, F9–F16, and Film 1–2 (mean ± SD, n = 3).
