# Supplementary material for: Development and In Vitro-Ex Vivo Evaluation of Novel Polymeric Nasal Donepezil Films for Potential Use in Alzheimer’s Disease Using Experimental Design
Source: Pharmaceutics. 2022 Aug 21;14(8):1742. doi: 10.3390/pharmaceutics14081742 (PMC9416078; doi:10.3390/pharmaceutics14081742)
Supplement: Supplementary file 1 [file pharmaceutics-14-01742-s001.zip › pharmaceutics-1856131-supplementary.pdf]

Development and In Vitro-Ex Vivo Evaluation of Novel Polymeric Nasal Donepezil Films for Potential Use in Alzheimer's Disease Using Experimental Design

Paraskevi Papakyriakopoulou [1], Dimitrios M. Rekkas [1], Gaia Colombo [2] and Georgia Valsami [1,*]

[1] Department of Pharmacy, School of Health Sciences, National and Kapodistrian University of Athens, 15784 Athens, Greece
[2] Department of Life Sciences and Biotechnology, University of Ferrara, 44121 Ferrara, Italy
* Correspondence: valsami@pharm.uoa.gr

Supplementary material

1. Film DH content

Method: The uniformity of drug content in the prepared nasal films was tested by determining the DH amount in different films of each formulation (round films, diameter=7.0 ± 0.55 mm, theoretical DH content 0.5 mg). In the case of formulations F8 and F17 the drug content was determined in the prepared gel as these compositions did not lead to film formation. Each film (or equivalent amount of gel for F8, F17) was dissolved in 10 mL of HPLC gradient water by sonication and then diluted with mobile phase. DH was measured in the diluted sample by HPLC-PDA, according to the method described in the section 2.3 of the main manuscript. Content uniformity test was performed six times for each formulation.

Results: The results of content uniformity of the 17 formulations and of the optimized one (Film 2) are presented in Table S1 and are expressed as % of the theoretical DH film content (mean ± SD).

Table S1. Film weight and content uniformity measurements expressed as % of the theoretical DH film content (mean ± SD, n=6).

Formulation	Film Weight	% content	AV (%) *
**F_1**	4.0 ± 0.08	94.3 (2.47)	11
**F_2**	6.9 ± 0.09	92.3 (1.80	11
**F_3**	2.0 ± 0.08	97.2 (2.37)	8
**F_4**	5.6 ± 0.07	95.9 (3.59)	12
**F_5**	1.0 ± 0.06	95.4 (3.43)	12
**F_6**	4.7 ± 0.07	90.0 (1.64)	14
**F_7**	3.9 ± 0.06	96.3 (3.34)	11
**F_8**	-	96.1 (2.90)	10
**F_9**	2.6 ± 0.09	93.4 (2.32)	12
**F_{10}**	4.2 ± 0.1	94.8 (3.02)	12
**F_{11}**	4.1 ± 0.15	95.7 (3.59)	12

**F_{12}**	3.4 ± 0.22	96.6 (3.96)	12
**F_{13}**	4.31 ± 0.18	96.7 (4.14)	12
**F_{14}**	3.2 ± 0.18	95.1 (3.02)	12
**F_{15}**	4.8 ± 0.19	95.5 (2.17)	9
**F_{16}**	2.6 ± 0.20	96.2 (3.01)	11
**F_{17}**	-	95.9 (3.29)	11
**Film 2**	2.9 ± 0.19	99.8 (4.89)	11

* AV is the Acceptance value, calculated using the equation: $AV = |M - \bar{X}| + ks$, where M is the reference value, $\bar{X}$ is the mean of individual contents, and ks is the acceptability constant [1].

2. Film thickness

Method: The thickness of the manufactured films was measured with INSIZE Outside Micrometer (Jiangsu, China) with a measuring range of 0-25 mm (0.001 mm graduation). The mean ± SD thickness values are presented vs HPMC E50 concentration (% w/w) in Figure S1.

Results: Figure S1 reveals the effect of the different concentrations of PEG 400 and Me-β-CD (0% to 3% and 0% to 6%, respectively) on film thickness when % HPMC E50 varies from 1% to 3%. Literature data on optimal thickness for pharmaceutical films, are limited and controversial. Generally, the ideal thickness for buccal films should fall between 50 to 1000 μm [2]. However, in the case of ocular films the limits are different, and the maximum accepted thickness is 90 μm, while a minimum is not specified [3]. In previous study, ocular films of thickness between 4.5 and 6.8 μm have been formulated, while it is also reported the development of nasal patches of thickness ranging from 190 to 390 μm [4]. In the present study, aiming to develop thin nasal films which do not interfere with breathing nor cause discomfort, 200 μm was set as upper acceptance limit. This limit derived from the preliminary experiments performed to select the factors' limits, and mainly by the thickness value (206.2 ± 4.6) measured for a formulation with the following composition of the film-forming solution: 4% HPMC E50, 3% PEG 400 and 6% Me-β-CD. This preliminary formulation had the plasticizer and permeation enhancer at their upper limits, but the HPMC concentration of 4% (w/v) resulted in a hard-to fold-film. As lower acceptance limit was chosen the lowest thickness value of films for drug administration found in the literature (4.5 μm).

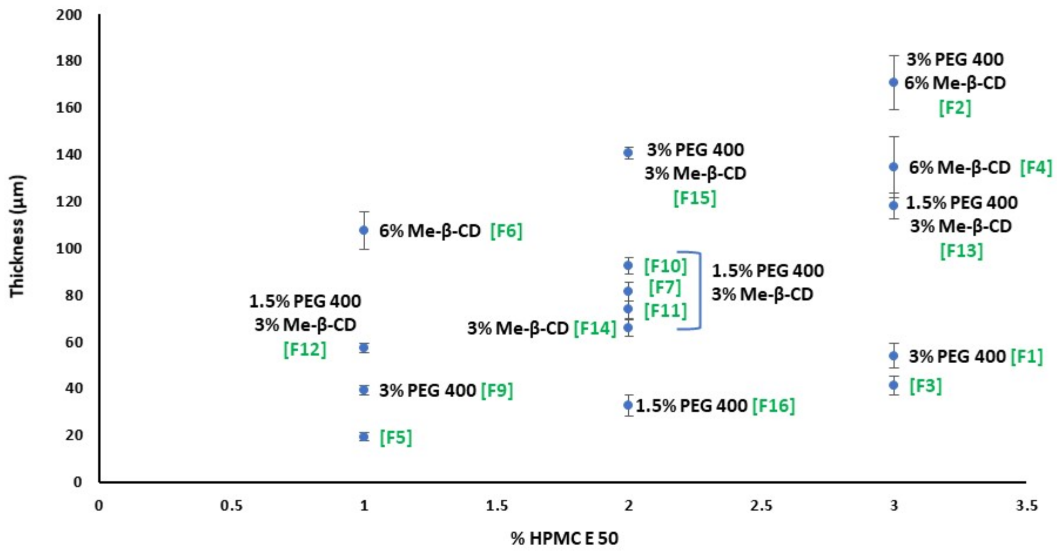

Figure S1. Thickness (mean ± SD, n=10) of manufactured nasal films as a function of the % concentration of HPMC E50 in the film-forming solution. Data labels indicate the % of PEG 400 and/or Me-β-CD, in the formulation.

1. Stability study in DH nasal films

Method: Stability study of the 17 film formulations, determined by the DoE, and of the optimized one (Film 2), was performed quantifying the DH content by HPLC, in round transparent films with diameter equal to 7.0 ± 0.55 mm. For film manufacturing, weighed amounts of the film-forming formulations (50 μL) were placed on the top of round blisters (Figure 1, main manuscript), by a Microman E, M250E, 50-250 μL pipette (Gilson, UK), and were let dry for 24 h at room temperature (25 °C). The dried films were stored at room temperature (25 °C), in airtight packaging. The DH amount on day 0 of the study was set as 100% (loading dose). At each time point of the stability study (1, 2, 3, 6 months and 1, 2, 3 months, for F1-F17 and Film 2, respectively), a triplet of each formulation was unpacked and examined for its appearance. Then, each film was placed in a 10 mL volumetric flask and dissolved in HPLC grade water. After sonication and appropriate dilutions, DH content in the films was assayed by HPLC-PDA as described in the section 2.3 of the main manuscript.

Results: The stability profiles over the 6 months period are presented in Figure S2 and the actual mean values (± SD) are shown in Table S2. The appearance of all the tested formulations remained the same during the entire stability study. However, a gradual decrease of DH content was observed in the case of F2 and F12, from the 90th day onwards. The HPLC chromatograms from the analysis of F12 films, during the stability study, included an additional peak, which can be due to a possible degradation product. Conversely, in the case of F2, DH quantification by HPLC-PDA showed a signal decrease, without any further peaks. However, further research is needed, to map the occurring molecular interactions between the three components and the API, the existence of which is also indicated by the results of the experimental design, included in the main manuscript.

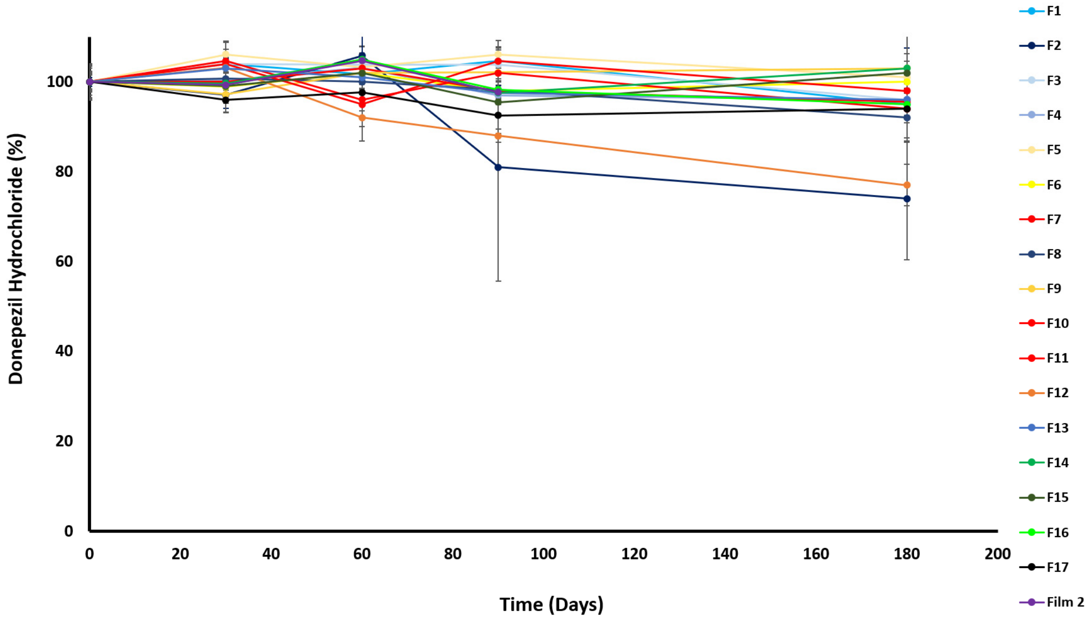

Figure S2. Stability profiles (mean ± SD, n=3) of the 6 month-stability study performed, in airtight packaging, in room temperature, on formulations F1-F17 and optimized formulation Film 2.

Table S2. Nasal film storage stability results, at 25 °C, presented as relative to day 0 % mean DH amount ± SD (n=3).

Formulation	Day30	Day60	Day90	Day180
	% of loading dose			
**F_1**	104 ± 2.0	102 ± 3.2	105 ± 1.0	95 ± 2.3
**F_2**	97 ± 4.0	106 ± 0.5	**81 ± 16.6**	**74 ± 13.4**
**F_3**	104 ± 1.0	104 ± 2.2	104 ± 3.2	96 ± 1.3
**F_4**	100 ± 4.2	103 ± 4.7	97 ± 1.4	96 ± 5.2
**F_5**	106 ± 1.1	103 ± 5.1	106 ± 2.9	101 ± 3.2
**F_6**	99 ± 2.1	102 ± 2.2	98 ± 3.9	100 ± 6.2
**F_7**	100 ± 4.8	103 ± 1.8	98 ± 1.3	96 ± 4.3
**F_8**	101 ± 2.4	100 ± 0.9	98 ± 1	92 ± 5.8
**F_9**	97 ± 1.1	102 ± 1.9	102 ± 1.9	103 ± 1.6
**F_{10}**	104 ± 1.4	95 ± 5.1	105 ± 3.0	98 ± 0.8
**F_{11}**	104 ± 1.3	96 ± 3.8	102 ± 2.5	94 ± 7.2
**F_{12}**	103 ± 5.6	92 ± 5.2	**88 ± 1.3**	**77 ± 4.6**
**F_{13}**	103 ± 6.1	101 ± 3.1	98 ± 4.6	96 ± 4.4
**F_{14}**	100 ± 2.7	105 ± 3.2	98 ± 2.0	103 ± 1.8
**F_{15}**	99 ± 3.4	102 ± 4.1	95 ± 0.4	102 ± 1.17
**F_{16}**	99 ± 5.9	105 ± 1.7	98 ± 1.7	95 ± 1.68
**F_{17}**	96 ± 3.0	98 ± 4.2	93 ± 3.8	94 ± 0.3
**Film 2**	101 ± 3.9	107 ± 5.0	100 ± 4.9	-

2. Percent (%) moisture loss

Methods: The percentage of moisture loss was determined in the 17 prepared formulations and in the optimized Film 2, in triplicate, applying Equation 1 (see main manuscript). The films were prepared following the process described in section 2.2, at the main manuscript. The dried films were detached, weighed (initial weight) and placed in the oven, at 40 °C. Then, they were re-weighed to determine any water loss and placed back in the oven. The same procedure was repeated at 48 h and 72 h (final weight). From 72 h onwards, no weight changes were observed for none of the tested formulation.

Results: Table S3 presents the % moisture loss values, expressed as mean ± SD (%). No values are reported for formulations F8, F17 because the film did not form, as described in the main manuscript. The tested nasal films present ≤ 3.0% moisture loss revealing the robustness and repeatability of the manufacturing process regarding the residual water in the pharmaceutical product.

Table S3. Percent (%) moisture loss of the formulations F1-F17 and optimized Film 2 (mean ± SD, n= 3).

Formulation	% moisture loss ± SD
**F_1**	0.8 ± 1.41
**F_2**	2.9 ± 0.05
**F_3**	0.9 ± 1.52
**F_4**	1.2 ± 1.02
**F_5**	1.5 ± 2.62
**F_6**	1.4 ± 1.22
**F_7**	1.7 ± 1.48
**F_8**	-
**F_9**	1.2 ± 2.14
**F_{10}**	1.6 ± 1.36
**F_{11}**	1.6 ± 1.41
**F_{12}**	3.0 ± 0.14
**F_{13}**	1.5 ± 1.31
**F_{14}**	1.1 ± 1.92
**F_{15}**	1.3 ± 2.31
**F_{16}**	1.2 ± 2.06
**F_{17}**	-
**Film 2**	1.1 ± 1.99

3. *In vitro* diffusion experiments and *ex vivo* permeation experiments

Methods: For the *in vitro* diffusion and *ex vivo* permeation experiments, either the artificial membrane or the extracted mucosa specimen were mounted between the donor and receptor compartments of the Franz cell. Cell equilibration, formulation loading into the donor, sampling, and recovering of residual DH from the donor, were carried out as described in sections 2.5 and 2.6 of the main manuscript. The drug

accumulated in the tissue was recovered according to the method described by Papakyriakopoulou et al. [5] and then quantified by HPLC, after centrifugation and appropriate dilutions. The DH amounts recovered from the mucosa, receptor and donor compartments allowed for the calculation of the mass balance.

The fluxes across the artificial membrane and nasal mucosa barrier to the receptor compartments were calculated from the slopes obtained from regression analysis of the respective amount of the drug permeated per unit area over the time, according to the equations 3 and 4 of the main manuscript.

Results: The fluxes of all the prepared formulations across the artificial and biological barriers are presented in Tables S4 and S5, respectively. The R-square values indicate the linearity of both release and permeation profiles, for all the prepared formulations. The quantification of DH remaining in the cellulose membranes revealed a negligible mean retention of the drug from the artificial membrane, equal to 1.6 ± 0.71 % of the loading doses of all the tested formulations. In the case of the nasal mucosa barrier, higher retention is observed, as it was expected due to the involvement of mucus into the drug-excipients network. The mean retention of the DH from the nasal mucosa was calculated equal to 10.1 ± 4.90 % of the loading doses of all the tested formulations. Membrane retention data of each formulation, expressed as the percent (%) of the loading dose retained by either the artificial or biological barrier are presented in Table S6.

Table S4. Flux (J) (mean ± SD, n=3) and R-square of the regression analysis of the amount of the drug permeated per unit area over the time, for formulations F1-F7, F9-F16, and Film 1-2.

Formulation	J (μg/cm²/min)	R^2
F_1	3.31 ± 0.042	0.9133
F_2	2.32 ± 0.024	0.9383
F_3	3.78 ± 0.040	0.9375
F_4	2.53 ± 0.012	0.9864
F_5	4.15 ± 0.043	0.9392
F_6	3.00 ± 0.033	0.9320
F_7	3.57 ± 0.022	0.9781
F_9	4.45 ± 0.063	0.8935
F_{10}	3.60 ± 0.028	0.9650
F_{11}	3.80 ± 0.027	0.9695
F_{12}	3.24 ± 0.014	0.9882
F_{13}	2.81 ± 0.025	0.9540
F_{14}	3.84 ± 0.030	0.9649
F_{15}	3.74 ± 0.032	0.9586
F_{16}	4.03 ± 0.061	0.8798
Film 1	4.60 ± 0.015	0.9934
Film 2	5.79 ± 0.056	0.9464

Table S5. Flux (J) (mean ± SD, n=3), R-square of the regression analysis of the amount of the drug permeated per unit area over the time and the apparent permeability (Papp) across the nasal mucosa barrier, for formulations F1-F7, F9-F16, and Film 1,2.

Formulation	J (µg/cm²/min)	R^2	Papp (cm/min) $*10^{-4}$
F_1	0.60 ± 0.021	0.9930	1.31
F_2	1.43 ± 0.068	0.9863	3.10
F_3	1.04 ± 0.069	0.9737	2.25
F_4	1.60 ± 0.041	0.9960	3.55
F_5	0.50 ± 0.037	0.9675	1.07
F_6	0.79 ± 0.058	0.9691	1.65
F_7	1.17 ± 0.028	0.9966	2.54
F_9	1.15 ± 0.061	0.9835	2.50
F_{10}	0.26 ± 0.038	0.8873	0.53
F_{11}	0.78 ± 0.039	0.9855	1.70
F_{12}	1.86 ± 0.040	0.9972	3.96
F_{13}	1.13 ± 0.000	0.9487	2.37
F_{14}	1.22 ± 0.056	0.9877	2.66
F_{15}	1.38 ± 0.000	0.9691	2.95
F_{16}	1.54 ± 0.000	0.9739	3.29
Film 1	1.22 ± 0.063	0.9840	2.59
Film 2	1.82 ± 0.000	0.9740	3.71

Table S6. Percent (%) of the loading dose retained by the cellulose membrane and the nasal mucosa barrier, of the formulations F1-F7, F9-F16, and Film 1-2 (mean ± SD, n= 3).

Formulation	% of the dose retained by the cellulose membrane ± SD	% of the dose retained by the nasal mucosa barrier ± SD
**F_1**	1.84 ± 0.69	11.5 ± 4.18
**F_2**	1.37 ± 0.98	15.5 ± 7.44
**F_3**	1.11 ± 0.45	13.2 ± 4.62
**F_4**	2.50 ± 1.67	12.9 ± 4.66
**F_5**	1.58 ± 1.08	10.2 ± 1.76
**F_6**	1.24 ± 0.36	12.5 ± 3.70
**F_7**	2.12 ± 1.23	10.8 ± 0.97
**F_9**	1.10 ± 0.87	12.7 ± 7.64
**F_{10}**	1.98 ± 0.25	9.9 ± 1.12
**F_{11}**	1.25 ± 0.83	11.2 ± 3.43
**F_{12}**	2.34 ± 0.56	15.8 ± 3.66
**F_{13}**	1.82 ± 0.55	10.3 ± 3.27
**F_{14}**	1.47 ± 0.34	16.0 ± 1.69
**F_{15}**	1.76 ± 1.64	12.8 ± 3.99
**F_{16}**	1.71 ± 0.34	1.7 ± 0.63
**Film 1**	0.17 ± 0.04	1.2 ± 0.33
**Film 2**	0.00 ± 0.00	1.5 ± 0.41

The results of formulations F8 and F17 are not presented in Tables S4-S6, as their composition does not allow the formation of film able to be detached from the blister.

References

1. Ph. Eur. 8.0, Chapter 2.9.40. Uniformity of dosage forms. 2012; 357-359.
2. Nair, A.B.; Kumria, R.; Harsha, S; et al. *In vitro* techniques to evaluate buccal films. *J. Controlled Release.* **2013**, 166(1), 10–21.
3. Tighsazzadeh, M.; Mitchell, J.C. Boateng JS. Development and evaluation of performance characteristics of timolol-loaded composite ocular films as potential delivery platforms for treatment of glaucoma. *Int. J. Pharm*. **2019**, 566, 111-125.
4. Laffleur, F. Nasal adhesive patches - Approach for topical application for dry nasal syndrome*. Int. J. Biol*. **2018**, 111, 493–497.
5. Papakyriakopoulou, P.; Manta, K.; Kostantini, C.; Kikionis, S.; Banella, S.; Ioannou, E.; Christodoulou, E.; Rekkas, D.M.; Dallas, P.; Vertzoni, M.; Valsami, G.; Colombo, G. Nasal powders of quercetin-β-cyclodextrin derivatives complexes with mannitol/lecithin microparticles for Nose-to-Brain delivery: *In vitr*o and *ex vivo* evaluation. *Int. J. Pharm*. **2021**, *607*, 121016.
